# P-28. Evaluation of oral antibiotic prescribing for uncomplicated Gram-Negative bloodstream infections following implementation of institutional guidance at an academic medical center

**DOI:** 10.1093/ofid/ofaf695.257

**Published:** 2026-01-11

**Authors:** Joshua Lechner, Mackenzie R Keintz, Jasmine R Marcelin, Molly M Miller, Elizabeth Lyden, Jihyun Ma, Trevor C Van Schooneveld, Scott J Bergman

**Affiliations:** Nebraska Medicine, North Liberty, Iowa; University of Nebraska Medical Center, Omaha, NE; University of Nebraska Medical Center, Omaha, NE; Nebraska Medicine, North Liberty, Iowa; University of Nebraska Medical Center, Omaha, NE; University of Nebraska Medical Center, Omaha, NE; University of Nebraska Medical Center, Omaha, NE; Nebraska Medicine, North Liberty, Iowa

## Abstract

**Background:**

Data have increasingly supported transition from intravenous (IV) to oral (PO) therapy for uncomplicated gram-negative bacteremia (uGNB). The purpose of this study was to evaluate differences in transition to PO therapy over time at our institution.

**Figure:**
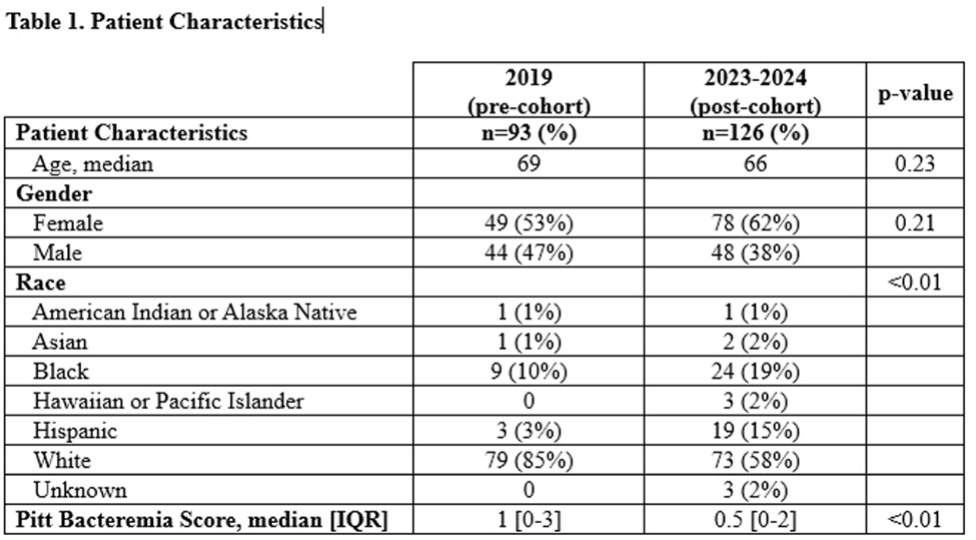

**Figure:**
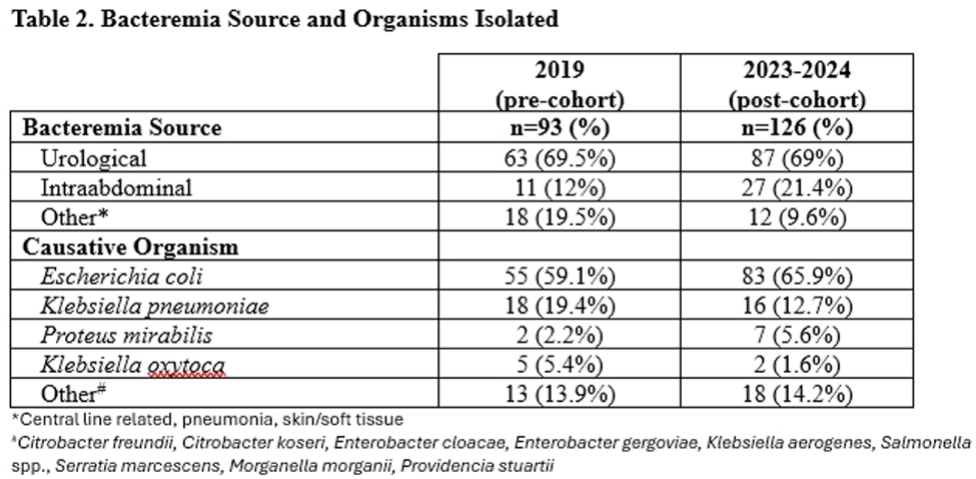

**Methods:**

Our antimicrobial stewardship team routinely reviews positive blood cultures and in September 2023 implemented guidance for management of uGNB. This was posted on our website and disseminated to clinicians, including hospitalists and ID physicians, encouraging best practices after susceptibilities return. Recommendations outlined selection criteria for PO therapy, preferred antibiotic regimens and total duration of 7 days. This retrospective, quasi-experimental study included immunocompetent adults at our 809-bed medical center with a first episode of uGNB during Jan-Dec 2019 (pre-cohort) compared to after guidance implementation (post-cohort, Nov 2023-Nov 2024). The primary outcome was proportion of patients transitioned to PO therapy for uGNB by day 7. Secondary outcomes included timing and choice of agent for IV to PO conversion and patient outcomes.

**Figure:**
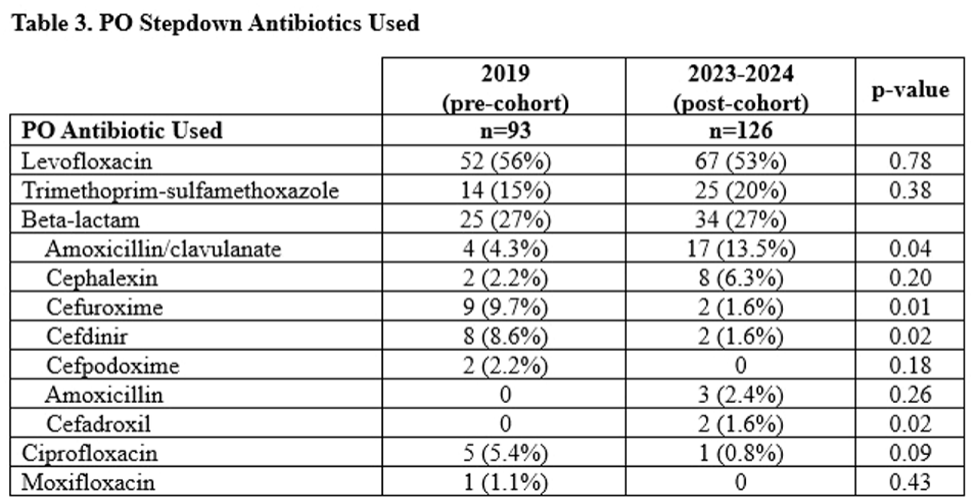

**Results:**

There were 93 patients in the pre-cohort and 126 patients in the post-cohort (Table 1). Most had urinary tract infections with *E.coli* (Table 2). Before guidance, 66.9% of patients were transitioned to PO antibiotics and after it was 71.2% (p=0.46). There was a significant decrease in median days of IV therapy (4 vs 3, p=< 0.01), length of hospital stay (6 vs 4 days, p=< 0.01) and total duration of antibiotics (10 vs 8 days, p=< 0.01). Levofloxacin was the most common PO stepdown in both cohorts (56% vs 53%, Table 3). Total use of beta-lactams was similar, but after implementation more patients were prescribed agents recommended by guidance including cephalexin (2.2% vs 6.3%) and amoxicillin/clavulanate (4.3% vs 13.5%, p=0.04). Agents listed as not recommended in guidance decreased, including cefdinir (8.6% vs 1.6%, p=0.02), cefpodoxime (2.2% vs 0%), and cefuroxime (9.7% vs 1.6%, p=0.01).

**Conclusion:**

Prescribing of PO stepdown antibiotic therapy for uGNB changed over time. After implementation of guidance, we observed significantly fewer days of IV antibiotics, shorter hospital stays, lower total durations, and increased use of recommended PO beta-lactams.

**Disclosures:**

Trevor C. Van Schooneveld, MD, FSHEA, FIDSA, BioMerieux: Advisor/Consultant|BioMerieux: Grant/Research Support

